# Human adenovirus DNA polymerase is evolutionarily and functionally associated with human telomerase reverse transcriptase based on *in silico* molecular characterization that implicate abacavir and zidovudine

**DOI:** 10.3389/fbinf.2023.1123307

**Published:** 2023-06-07

**Authors:** Toluwase Hezekiah Fatoki

**Affiliations:** Applied Bioinformatics Laboratory, Department of Biochemistry, Federal University Oye-Ekiti, Oye, Nigeria

**Keywords:** human adenoviruses (HAdVs), hypothetical proteins, antiretroviral drugs, DNA polymerase, molecular dynamics and docking

## Abstract

Human adenoviruses (HAdVs) are non-enveloped, small double stranded DNA (dsDNA) viruses that cause asymptomatic infections, clinical syndromes and significant susceptibility to infections in immunocompromised people. The aim of the present study was to identify critical host proteins and HAdV hypothetical proteins that could be developed as potential host-viral targets for antiHAdV therapy. Here, the function of selected hypothetical proteins of HAdV based on phylogenetic relationship with the therapeutic targets of antiretroviral drugs of human immunodeficiency virus (HIV) was predicted computationally, and characterized the molecular dynamics and binding affinity of DNA polymerase of HAdV. Thirty-eight hypothetical proteins (HPs) of human adenovirus (HAdV) were used in this study. The results showed that HAdV DNA polymerase (P03261) is related to Human TERT (O14746) and HLA-B (P01889) genes. The protein-protein interaction of human five molecular targets (PNP, TERT, CCR5, HLA-B, and NR1I2) of ARVDs are well-coordinated/networked with CD4, AHR, FKBP4, NR3C1, HSP90AA1, and STUB1 proteins in the anti-HIV infection mechanism. The results showed that the free energy score of abacavir and zidovudine binding to HAdV DNA polymerase are −5.8 and −5.4 kcal mol^-1^ respectively. Also, the control drug, cidofovir and ganciclovir have less binding affinity for DNA polymerase of HAdV when compare to that of abacavir and zidovudine. Similarity was observed in the binding of abacavir and zidovudine to HAdV DNA polymerase (ASP742, ALA743, LEU772, ARG773 and VAL776). In conclusion, combination of abacavir and zidovudine was predicted to be potential therapy for controlling HAdV infection targeting HAdV DNA polymerase.

## Introduction

Human adenoviruses (HAdVs) are non-enveloped, small double stranded DNA (dsDNA) viruses that cause asymptomatic infection and clinical syndromes, including hepatitis, myocarditis, gastroenteritis, pneumonia, upper respiratory tract infection, conjunctivitis, and infections of the urinary tract (Curlin et al., 2010; Robinson et al., 2013; Naveed et al., 2017). Globally, about 5%–7% of respiratory tract infections has been attributed to HAdV in pediatrics population below age of 5 years, and that HAdVs cause significant susceptibility to infections in immunocompromised people (Hierholzer, 1992; James et al., 2007; Ghebremedhin, 2014; Scott et al., 2016). Adenoviruses can cause life-threatening infections in hematopoietic stem cell transplant recipients, including hepatitis, pneumonitis, colitis, nephritis, and encephalitis (La Rosa et al., 2001).

The adenovirus has a linear genome of about 35 kBp in size with a 55 kDa the terminal protein (TP) covalently attached to each 5’ end. The 140 kDa adenovirus-encoded DNA polymerase (Ad Pol) is required for viral DNA replication both *in vitro* and *in vivo* (Field et al., 1984). The *in vitro* replication of adenovirus DNA using HeLa cell, requires at three proteins from the virus (the 59 kDa DNA-binding protein (DBP), the 80 kDa precursor TP, and the 140 kDa Ad Pol); and two proteins from the host (HeLa cell) are nuclear proteins (factor I and factor II**)**, which are both required for initiation and elongation of viral DNA synthesis (Field et al., 1984). A study has shown that nuclear factor II of HAdV possesses type I topoisomerase activity and could be substituted in the *in vitro* DNA replication system with purified type I topoisomerases from HeLa cells or calf thymus but not that *Escherichia coli* (Nagata et al., 1983).

Currently, there is no Food and Drug Administration (FDA) approved drug for the treatment of adenovirus infection, but the available regimen are the combinations of existing antiviral drugs such as cidofovir and its derivative (3-hexadecyloxy-1-propanol-cidofovir (also known as brincidofovir or CMX001)), ganciclovir, filociclovir, cytarabine, ivermectin, zalacitabine, arbidol (umifenovir), stavudine and ribavirin as well as anticancer drugs such as camptothecin, and other chemicals such as epiandrosterone (Wong and Hsu, 1990; Waye and Sing, 2010; Kumar et al., 2019; Saha and Parks, 2020; Dodge et al., 2021), RIDK34, digitoxigenin and digoxin (Grosso et al., 2017), Tazarotene (Wang et al., 2018) and Verdinexor (Widman et al., 2018).

Moreover, HAdVs have wide applications as vector systems for drug delivery and vaccines development (such as human immunodeficiency virus (HIV), malaria, influenza, tuberculosis, and Ebola virus) due to their ability to infect a range of mammalian host cells, flexibility to accommodate substantial genetic recombinant inserts, and excellent safety profile (Curlin et al., 2010). Molecular associations have been established between HIV, HAdV and hepatitis B virus (HBV), and the correlation of molecular targets of antiretroviral drugs (ARVDs) with the HPs of HAdV might open avenues for prioritizing novel drug targets (Ghebremedhin, 2014; Kolawole et al., 2014; Singh et al., 2017).

A deeper understanding of metabolic impact of the existing antiretroviral drugs (ARVDs) in the host cell gene expression at the protein and RNA levels could be used to infer treatment, for posttranscriptional regulation in adenovirus-infected cells (Zhao et al., 2017). The aim of the present study was to identify critical host proteins and HAdV hypothetical proteins that could be developed as potential host-viral targets for anti-HAdV therapy. Here, the function of selected hypothetical proteins of HAdV based on phylogenetic relationship with the therapeutic targets of antiretroviral drugs of human immunodeficiency virus (HIV) was predicted computationally, and characterized the molecular dynamics and binding affinity of DNA polymerase of HAdV.

## Methods

### HAdV hypothetical protein sequence analysis

The list of 38 hypothetical proteins (HPs) of human adenovirus (HAdV) along with their Uniprot ID, corresponding genome and protein length were obtained from the literature (Naveed et al., 2017), and their sequences were obtained from UniProt database. the sequence properties such as isoelectric point, net charge, extinction coefficients and improbability of expression in inclusion bodies, were analyzed on EMBOSS Pepstats (www.ebi.ac.uk/Tools/seqstats/emboss_pepstats).

### Antiretroviral drugs and their molecular targets preparation

The structures of the antiretroviral drugs (ARVDs) were obtained from NCBI PubChem Compound database (https://pubchem.ncbi.nlm.nih.gov/) in SMILES, and they were reconstructed and subjected to 3D structure optimization using ACDLabs/ChemSketch software, and the structures were saved in MOL format. File conversion from MOL format to PDB format was done using PyMoL v2.0.7. The molecular targets of these antiretroviral drugs were obtained from DrugBank database (https://go.drugbank.com/drugs/).

### Phylogenetic analysis and structural modeling

The phylogenetic analysis of the HAdV hypothetical proteins with and without the molecular targets of the ARVDs, were done using ClustalO server (www.ebi.ac.uk/tools/clustalo). The structure of selected one HAdV and HBV proteins were modelled using SwissModel server: https://swissmodel.expasy.org/(Waterhouse et al., 2018; Studer et al., 2021). The modeled structure was analyzed using SAVES webserver v6.0 (http://saves.mbi.ucla.edu), tools such as ERRAT and PROCHECK, were used to check for the error function and stereochemical quality of the modelled protein structure. Based on the phylogenetic result, the sequence comparison of HAdV DNA polymerase and Human TERT and HLA-B target proteins of ARVD, were done on EMBOSS DotMatcher (www.ebi.ac.uk/Tools/seqstats/emboss_dotmatcher).

### Human host metabolic genes network

It is important to appreciate the intracellular metabolism of the ARVDS. The gene IDs of the predicted target proteins for the ARVDs were compiled and used for expression network analyses **(**transcription factor enrichment analysis and protein-protein interaction network expansion and kinase enrichment analysis), using eXpression2Kinases (X2K) Web server https://maayanlab.cloud/X2K/(Clarke et al., 2018), where human was selected as background organism.

### ARVDs human targets protein-protein interaction analysis

The gene ID of ARVDS molecular target proteins in humans and HPs of HAdV, were respectively analyzed for protein-protein interaction (PPI) profile on the STRING webserver (https://string-db.org/, Szklarczyk et al., 2021).

### Molecular docking studies

The molecular docking studies were carried out according to the method of Fatoki et al. (2022), using the ARVDs that have multiorganism (HIV, HBV or Human) targets based on documented reports on drugbank database as shown in Table 1, where cidofovir (DB DB00369) and ganciclovir (DB01004) were used as control. Briefly, the target proteins and ligands were prepared for docking using AutoDock Tools (ADT) v1.5.6 (Morris et al., 2009) at default settings, with only polar hydrogen atoms, Kollman charges and Gasteiger charges added, and the output file was saved in pdbqt format. Molecular docking program AutoDock Vina v1.2.3 (Trott and Olson, 2010; Eberhardt et al., 2021) was employed to perform the active site docking experiment. After docking, close interactions of binding of the target with the ligands were analyzed and visualized on PyMol and PoseView webserver available at https://proteins.plus/(Stierand et al., 2006).

**TABLE 1:** Antiretroviral drugs information and molecular targets.

SN	Class	Drug information			Drug Impact		
		Drug name	PubChem ID	Drugbank ID	Metabolism truck (gene name)	Molecular target	
						Name	UniProt ID
1	Nucleoside/nucleotide reverse transcriptase inhibitors (NRTIs)	Lamivudine	60825	DB00709	DCK, CMPK1, PGK1, NME1, NME2, ALB, PCYT1A, PCYT2, NT5C, ABCC1, SLC22A6, ABCG2, SLC22A1, SLC22A2, SLC22A3, ABCB1, ABCC4, ABCC3, ABCC2	Reverse transcriptase/RNaseH	Q72547
						Protein P (HBV-F)	Q05486
		Tenofovir Disoproxil	5481350	DB00300	AK2, AK4, SLC22A6, SLC22A8, ABCC10, ABCC4, ABCC2, ABCB1, NME1, CKB	Reverse transcriptase/RNaseH (HIV)	Q72547
						DNA polymerase (HBV-D)	P24024
		Zidovudine	35370	DB00495	CYP2A6, CYP2C9, CYP3A4, ALB, TK1, ABCB1, UGT2B7, UGT1A1, SLC22A2, SLC22A6, SLC22A7, SLC22A8, SLC22A11, SLC28A1, SLC29A2, ABCC4, ABCC5, ABCG2	Reverse transcriptase/RNaseH	Q72547
						Telomerase reverse transcriptase (Human)	O14746
		Abacavir	441300	DB01048	ADK, ADH6, UGT1A1	Reverse transcriptase/RNaseH	Q72547
						HLA class I histocompatibility antigen, B-57 alpha chain (Human)	P01889
		Didanosine	135398739	DB00900	ALB, SLC22A6, SLC29A1, SLC29A2	Reverse transcriptase/RNaseH	Q72547
						Purine nucleoside phosphorylase (Human)	P00491
		Stavudine	18283	DB00649	ALB, SLC22A6, SLC28A1	Reverse transcriptase/RNaseH	Q72547
		Emtricitabine	60877	DB00879	DCK, ALB, SLC47A1	Reverse transcriptase/RNaseH	Q72547
2	Integrase inhibitors	Dolutegravir	54726191	DB08930	UGT1A1, UGT1A3, CYP3A4, YP3A45, UGT1A9, SLC22A2, ABCG2, SLC22A6, SLC22A8, SLC47A1, ALB.	Integrase	Q7ZJM1
		Raltegravir	54671008	DB06817	UGT1A1	Integrase	Q7ZJM1
		Cabotegravir	54713659	DB11751	UGT1A1, UGT1A9, ALB, SLC22A6, SLC22A8, ABCG2, ABCB1	Integrase	Q7ZJM1
		Elvitegravir	5277135	DB09101	CYP3A4, UGT1A1	Integrase	Q7ZJM1
		Bictegravir	90311989	DB11799	CYP3A4, UGT1A1, POU2F2, SLC47A1	Integrase	Q7ZJM1
						Reverse transcriptase/RNaseH	Q72547
3	Non-nucleoside reverse transcriptase inhibitors (NNRTIs)	Doravirine	58460047	DB12301	CYP3A4, CYP3A5	Reverse transcriptase/RNaseH	Q72547
		Efavirenz	64139	DB00625	CYP2C19, CYP2C9, CYP2B6, CYP3A4, CYP3A5, CYP3A7, CYP1A2, CYP2D6, CYP2C8, UGT1A1, ALB, ABCB11, SLC22A1	Reverse transcriptase/RNaseH	Q72547
		Nevirapine	4463	DB00238	CYP2D6, CYP1A2, CYP3A7, CYP3A4, CYP2B6, CYP3A5, CYP2C9, CYP2A6, ALB, SLC22A1	Reverse transcriptase/RNaseH	Q72547
		Delavirdine	5625	DB00705	CYP3A4, CYP2D6, CYP3A5, CYP3A7, CYP2C9, CYP2C19	Reverse transcriptase/RNaseH	Q72547
		Etravirine	193962	DB06414	CYP3A4, CYP2C9, CYP2C19, ABCB1, ABCB4	Reverse transcriptase/RNaseH	Q72547
						Gag-Pol polyprotein	P04585
		Rilpivirine	6451164	DB08864	CYP3A4, CYP2C19, CYP2B6, CYP2D6, CYP2C9, CYP2C8, CYP2E1, ALB, ABCB1, ABCG2, SLCO1B1, SLCO1B3	Reverse transcriptase/RNaseH	Q72547
						Nuclear receptor subfamily 1 group I member 2 (Human)	O75469
4	Protease inhibitors	Ritonavir	392622	DB00503	CYP3A4, CYP2D6, CYP2C9, CYP2C19, CYP2B6, CYP2C8, CYP1A2, CYP3A5, CYP3A7, ALB, ORM1, ABCB1, ABCC1, SLCO1A2, ABCC2, ABCG2, SLCO1B1, SLCO2B1, ABCB11, SLCO1B3	Human immunodeficiency virus type 1 protease (Pol polyprotein)	Q72874
						Nuclear receptor subfamily 1 group I member 2 (Human)	O75469
		Atazanavir	148192	DB01072	CYP3A4, CYP2C9, UGT1A1, CYP2C8, CYP1A2, ABCC1, SLCO1B1, ALB, SLCO1B3, ABCB11, SLCO2B1, ABCB1	Human immunodeficiency virus type 1 protease (Pol polyprotein)	Q72874
		Darunavir	213039	DB01264	CYP2D6, CYP3A4, ALB, ORM1, ABCB1, SLCO1B1	Human immunodeficiency virus type 1 protease (Pol polyprotein)	Q72874
		Amprenavir	65016	DB00701	CYP2B6, CYP2C19, CYP2C9, CYP2D6, CYP3A5, CYP3A4, ABCB1, ABCC1, SLCO1B1	Human immunodeficiency virus type 1 protease (Pol polyprotein)	Q72874
		Tipranavir	54682461	DB00932	CYP3A4, CYP2C9, CYP1A2, CYP2D6, CYP2C19, UGT1A1, OATP1B1/SLCO1B1, BSEP/ABCB11, ABCB1	Human immunodeficiency virus type 1 protease (Pol polyprotein)	Q72874
		Indinavir	5362440	DB00224	CYP2D6, UGT1A1, CYP3A4, CYP3A5, CYP3A7, ABCB1, SLC22A1, ABCC1, SLCO1A2, SLCO1B1, ABCC2, SLCO2B1, ABCB11	Human immunodeficiency virus type 1 protease (Pol polyprotein)	Q72874
		Saquinavir	441243	DB01232	CYP3A4, CYP3A5, CYP3A7, CYP2C8, CYP2D6, OCT1, OATP1B1/SLCO1B1, ABCB1, ORM1, ALB, SLC22A1, SLCO2B1, ABCB11, SLCO1A2, ABCG2, ABCC1, ABCC2	Human immunodeficiency virus type 1 protease (Pol polyprotein)	Q72874
		Lopinavir	92727	DB01601	CYP3A4, CYP2D6, CYP1A2, CYP2C19, CYP2B6, CYP2C9, ORM1, ALB, ABCB1, SLCO1B1, SLCO1B3, ABCB11	Human immunodeficiency virus type 1 protease (Pol polyprotein)	Q72874
		Nelfinavir	64143	DB00220	CYP3A4, CYP3A7, CYP3A5, CYP2B6, CYP2C19, UGT1A1, CYP2C9, CYP2D6, ALB, ORM1, ABCB1, SLC22A1, SLCO1A2, ABCG2, SLCO1B1, SLCO1B3, SLCO2B1, ABCB11	HIV-1 protease	O90777
5	Entry/Attachment Inhibitors	Maraviroc	3002977	DB04835	CYP3A4	C-C chemokine receptor type 5 (Human)	P51681
		Enfuvirtide	16130199	DB00109	CYP2C19, CYP2E1	Envelope glycoprotein	Q53I07
		Fostemsavir	11319217	DB11796	CYP3A4, ABCB1, ABCG2, SLCO1B1, ALB, SLCO1B3, UGT1A1	Envelope glycoprotein gp160	P12488
6	Pk Enhancers (Boosters)	Cobicistat	25151504	DB09065	CYP3A4, CYP3A5, CYP3A7, CYP3A43, CYP2D6, ABCB1, ABCG2, SLCO1B1, SLCO1B3	None	none

### Protein molecular dynamics simulation

The flexibility dynamics of HAdV DNA Pol proteins from the PPI were analyzed using CABSflex2 server (http://biocomp.chem.uw.edu.pl/CABSflex2/) to evaluate the root mean square fluctuation (RMSF) of the structures. Small- and wide-angle X-ray scattering (SWAXS) curves based on explicit-solvent all-atom molecular dynamics (MD) simulations of the selected core proteins were analyzed on WAXSiS server: http://waxsis.uni-goettingen.de (Knight and Hub, 2015) to assess average value of radius of gyration (Rg).

### Protein-ligand molecular dynamics simulation

Molecular dynamics simulations were performed for 100 nanoseconds using Desmond, a Package of Schrödinger LLC (Bowers et al., 2006; Schrödinger, 2018; Fatoki et al., 2023). The initial stage of protein and ligand complexes for molecular dynamics simulation were obtained from docking studies. The protein–ligand complexes were preprocessed using maestro’s protein preparation wizard, which also included optimization and minimization of complexes. All systems were prepared by the System Builder tool. Solvent Model with an orthorhombic box was selected as TIP3P (Transferable Intermolecular Interaction Potential 3 Points). The Optimized Potential for Liquid Simulations (OPLS)-2005 force field was used in the simulation (Shivakumar et al., 2010). The models were made neutral by adding counter ions 0.15 M NaCl to mimic the physiological conditions (Fatoki, 2022). The NPT ensemble (Isothermal-Isobaric: moles (N), pressure (P), and temperature (T) are conserved) with 300 K temperature and 1 atm pressure was select for complete simulation. The models were relaxed before the simulation. The trajectories were saved after every 100 ps during simulation, and post-simulation analysis of the trajectories were done to determine the root-mean-square deviation (RMSD), root-mean-square fluctuation (RMSF), radius of gyration (Rg), solvent accessibility surface area (SASA), protein-ligand interaction profile. Also, prime molecular mechanics/generalized Born surface area (MMGBSA) was evaluated and binding free energy was calculated as follows:

$$•MMGBSA\;\Delta G^{bind}=\Delta G^{complex}-\Delta G^{protein}-\Delta G^{ligand}$$

$$•MMGBSA\;\Delta G^{bind}=\Delta G^{Coulomb}+\Delta G^{Covalent}+\Delta G^{Hbond}+\Delta G^{Lipo}+\Delta G^{Packing}+\Delta G^{SolvGB}+\Delta G^{vdW}$$

where $\Delta G^{bind}$ is the total Prime energy, Hbond denote hydrogen bonding energy, Lipo is lipophilic energy, Packing represents pi-pi packing correction. SolvGB is generalized Born electrostatic solvation energy, and vdW is Van der Waals energy (Zhang et al., 2017; Schrödinger, 2019).

## Results

Thirty-eight hypothetical proteins (HPs) of human adenovirus (HAdV) were used in this study. Among these 38 HPs, only HAdV-2 protein P03261 has the largest amino acid residues (aa.1198) with isoelectric point near neutrality (pH 7.4) as shown in Supplementary Table S1. The molecular targets of 30 ARVDs obtained are classified into three based on organism status: human (telomerase reverse transcriptase (hTERT) [UniProt ID: O14746], HLA class I histocompatibility antigen, B-57 alpha chain (HLA-B) [UniProt ID: P01889]), purine nucleoside phosphorylase (PNP) [UniProt ID: P00491], nuclear receptor subfamily 1 group I member 2 (NR1I2) (UniProt ID: O75469) and C-C chemokine receptor type (CCR5) [UniProt ID: P51681]); hepatitis B visus (protein P [UniProt ID: Q05486] and DNA polymerase (UniProt ID: P24024]); and human immunodeficiency virus (reverse transcriptase/RNaseH [UniProt ID: Q72547], integrase [UniProt ID: Q7ZJM1], Gag-Pol polyprotein [UniProt ID: P04585], HIV-1 protease (Pol polyprotein) [UniProt ID: Q72874; O90777], and envelope glycoprotein [UniProt ID: Q53I07, P12488]), as shown in Table 1.

**FIGURE 1:**
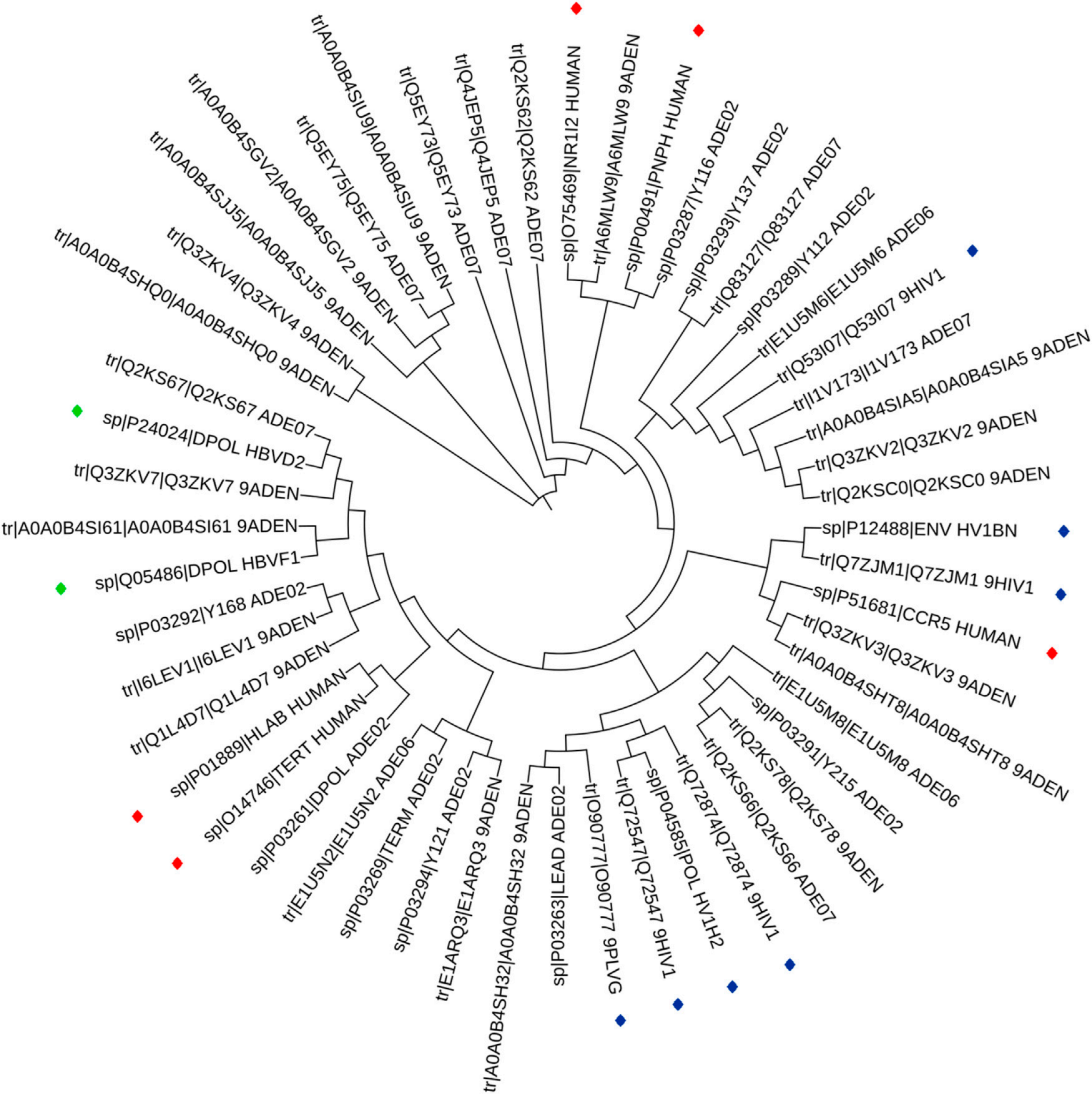
Phylogentic tree of the 14 ARVDs molecular targets (colored stars) together with 38 hypothetical proteins of HAV.

**FIGURE 2:**
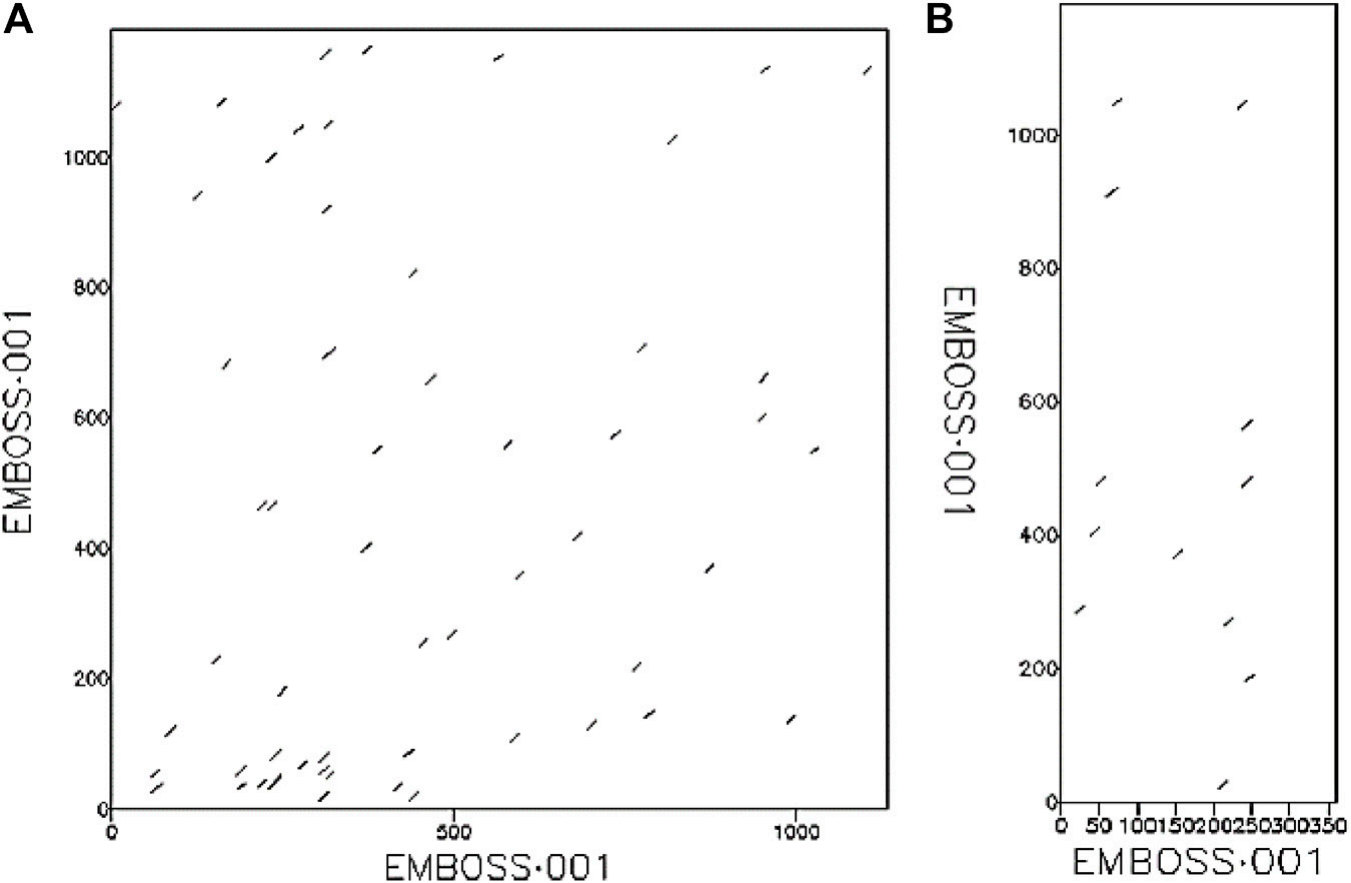
The relationship between **(A)** HAdV DNA Pol protein (UniProt ID: P03261) and human TERT protein (UniProt ID: O14746), **(B)** HAdV DNA Pol protein (UniProt ID: P03261) and human HLA-B protein (UniProt ID: P01889).

The phylogenetic results in Figure 1, showed that HAdV proteins Q2KS67, Q3ZKV7, and A0A0B4SI61 are related to HBV DNA polymerase (P24024 and Q05486); P03261 is related to Human TERT (O14746) and HLA-B (P01889) genes; Q3ZKV3 is related to human CCR5 protein (P51681); A6MLW9 is related to human NR1I2 (O75469) and PNP (P00491) proteins. A0A0B4SH32 and P03263 are related to HIV-1 protease (O90777). I1V173, A0A0B4SIA5, Q3ZKV2 and Q2KSC0 are related to HIV-1 envelope glycoprotein (Q53I07) in functions. The relationship between HAdV (P03261) and two human proteins (TERT, O14746 and HLA-B, P01889) is shown in Figure 2, where greater similarity was found between HAdV (P03261) and hTERT (O14746).

**FIGURE 3:**
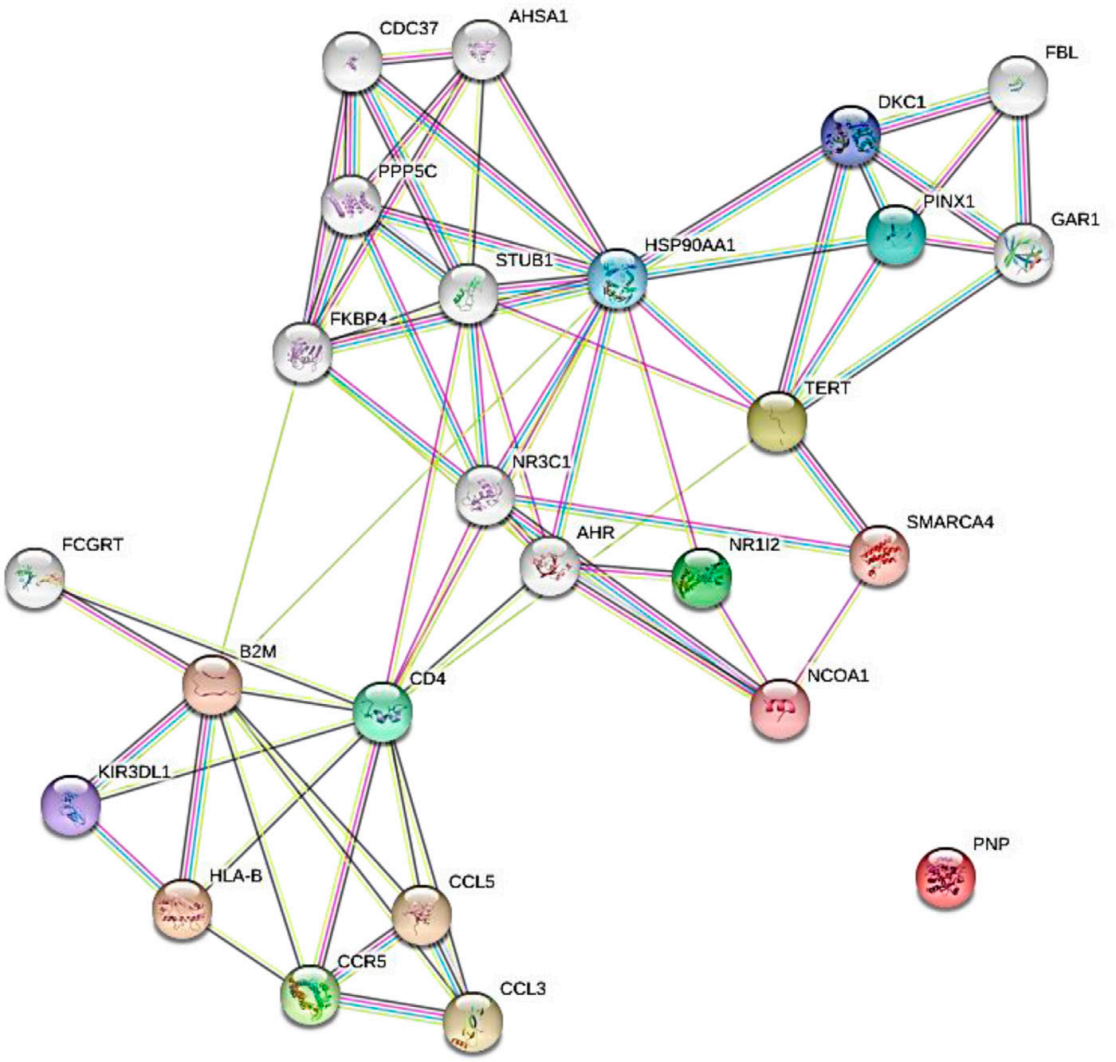
Expanded protein-protein interaction of five molecular targets (PNP, TERT, CCR5, HLA-B, and NR1I2) of ARVDs in human.

**FIGURE 4:**
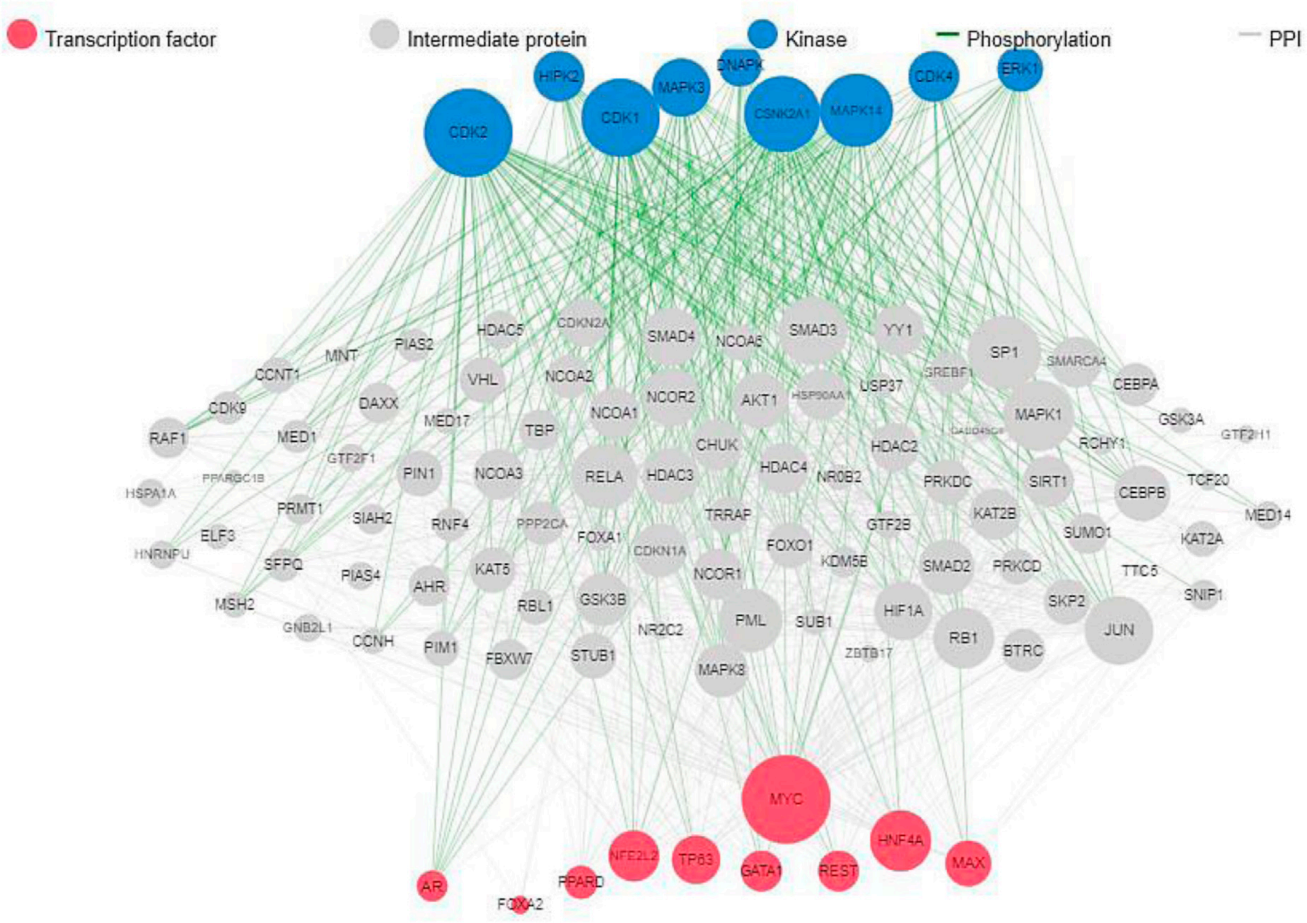
Gene network interactions of human metabolic genes impacted by ARVDs.

The results of protein-protein interaction of human five molecular targets (PNP, TERT, CCR5, HLA-B, and NR1I2) of ARVDs are well-coordinated/networked with CD4, AHR, FKBP4, NR3C1, HSP90AA1, and STUB1 proteins in the anti-HIV infection mechanism (Figure 3). The gene network analysis of the metabolism genes involved in ARVD reveal the role of transcription factors (MYC, HNF4A, MAX, TP63, GATA1, REST, PPARD, FOXA2, and NFE2L2) and kinases (CDK1, CDK2, CDK4, HIPK, DNAPK, MAPK3 and MAPK14) in antiviral mechanism (Figure 4).

The model structure of HAdV DNA polymerase (HAdV DNa pol) was done on swiss-modelling server using Phi29 DNA polymerase (PDB ID: 1XHX) with resolution of 2.35Å, which has sequence identity and similarity of 19.58 and 0.29 respectively, in a range of 410-825 amino acid residues, forming a monomeric structure. The model on Swissmodel showed MolProbility score of 1.90 and Ramachandra favoured 83.60% of the amino acids. The overall quality factor of the model by ERRAT plot was 81.70% (Supplementary Figure S1) while the Ramachandran plot generated by PROCHECK showed that 80.5% of the amino acid residues are in the most favoured region, 16.5% in the allowed region, 1.8% in the general region, and 1.3% in the disallowed region. This model structure of HAdV DNa pol was also used for molecular dynamic simulation and docking analyses.

**FIGURE 5:**
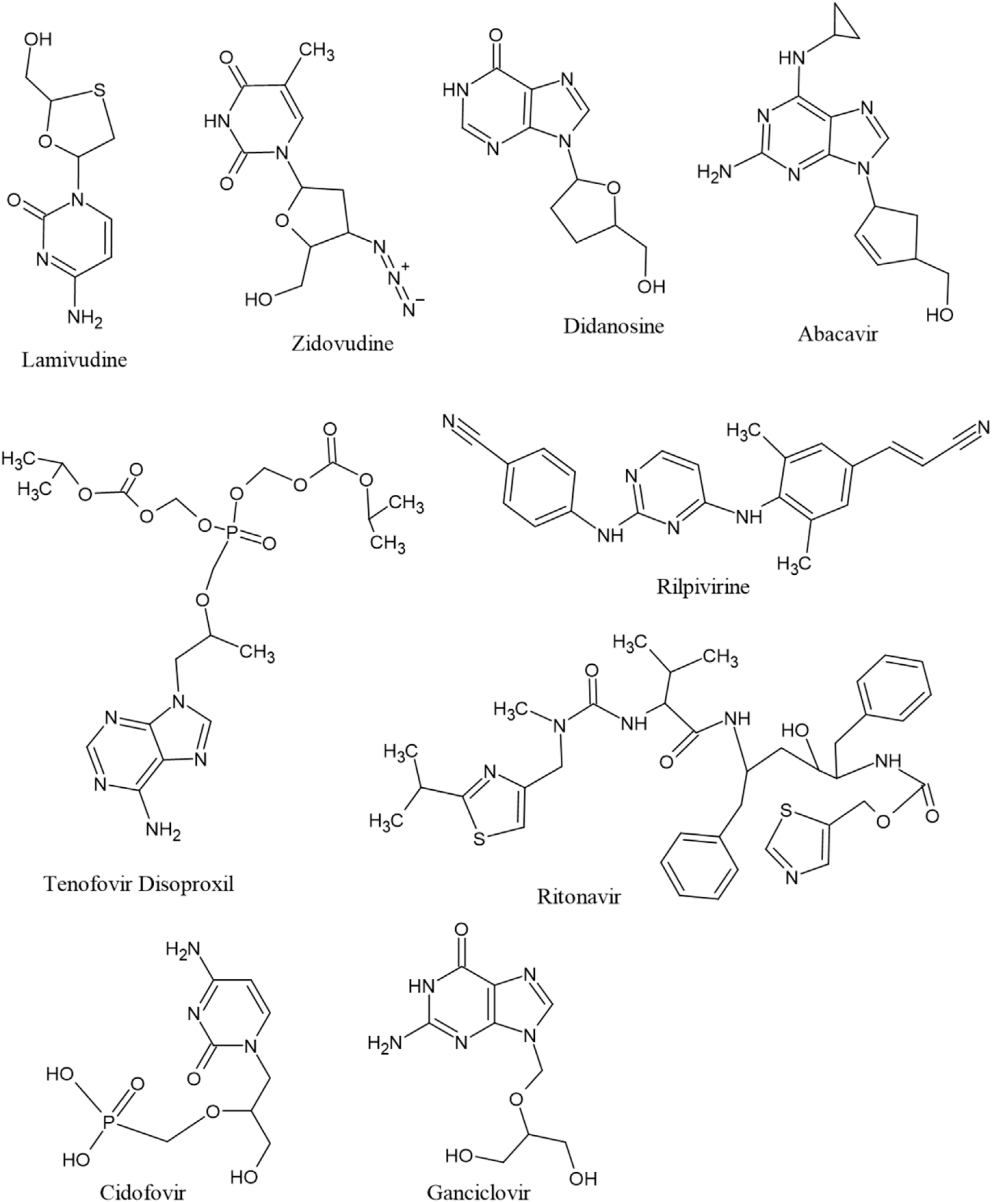
Structure of seven multitargeted anti-HIV drugs together with two anti-HHV drugs (cidofovir and ganciclovir).

**FIGURE 6:**
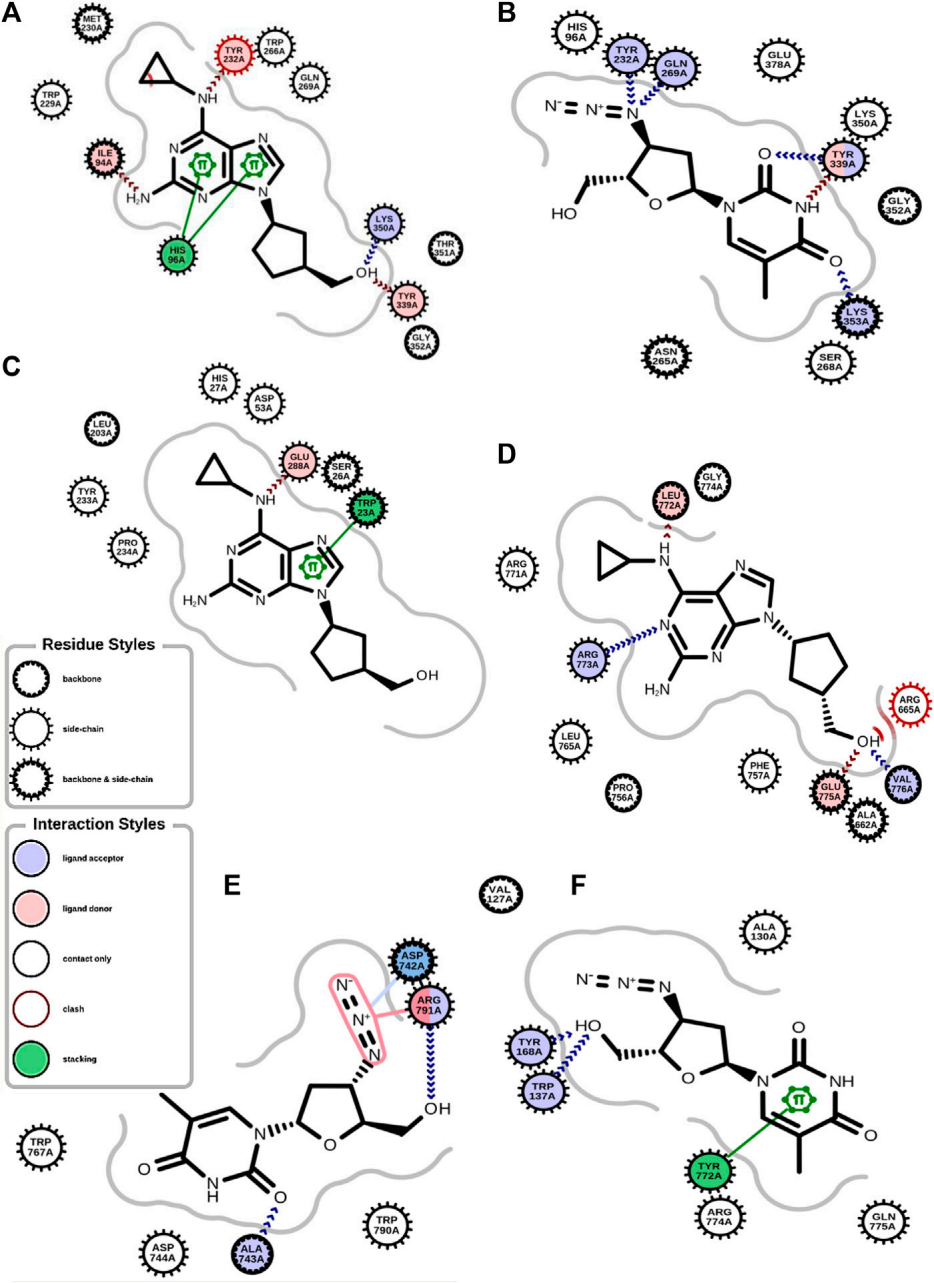
2D structure of binding interaction of **(A)** 2JLE-Abacavir complex. **(B)** 2JLE-Zidovudine complex. **(C)** AF-P01889-Abacavir complex. **(D)** Model_P03261-Abacavir complex. **(E)** Model_P03261-Zidovudine complex. **(F)** AF-O14746-Zidovudine complex.

**FIGURE 7:**
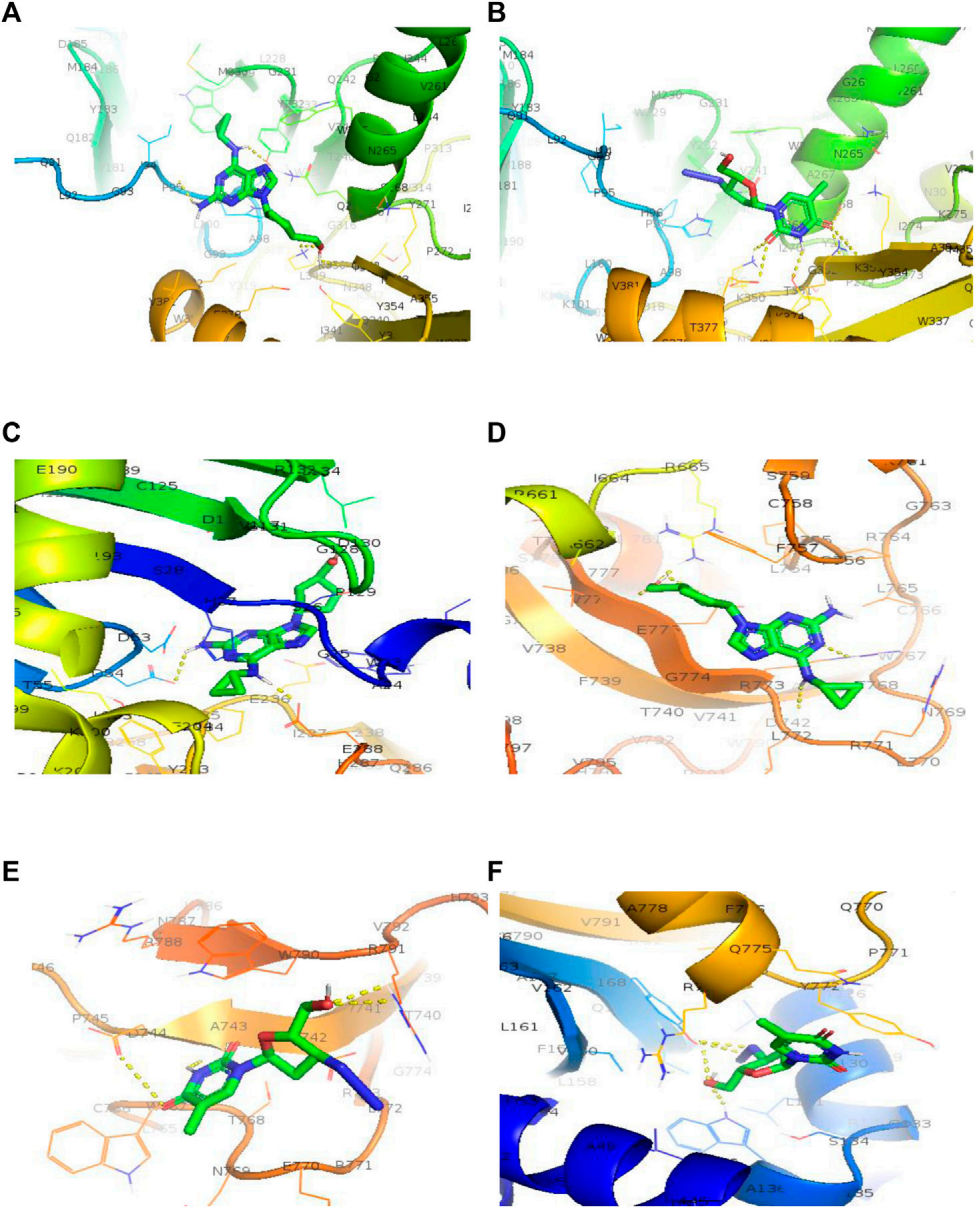
3D structure of binding interaction of **(A)** 2JLE-Abacavir complex. **(B)** 2JLE-Zidovudine complex. **(C)** AF-P01889-Abacavir complex. **(D)** Model_P03261-Abacavir complex. **(E)** Model_P03261-Zidovudine complex. **(F)** AF-O14746-Zidovudine complex.

The antiviral drugs that met the criteria of multi-organismal targets were used for molecular docking study and their chemical structures are shown in Figure 5. Molecular docking studies provide a prediction of ligand binding status in static conditions. Molecular docking of HAdV DNA polymerase was done based on the results of phylogenetic analysis, and the results are shown in Table 2. The results showed that the free energy score of abacavir and zidovudine binding to HAdV DNA polymerase (−5.8 and −5.4 kcal mol^-1^ respectively) are less than that of HIV reverse transcriptase/RNaseH (−6.5 and −6.2 kcal mol^-1^ respectively), human telomerase reverse transcriptase (hTERT) (−6.2 kcal mol^-1^) and human HLA class I histocompatibility antigen, B-57 alpha chain (−6.3 kcal mol^-1^). The 3D structures of some of the binding interactions are shown in Figure 6, while the 2D structures are indicated in Figure 7. Also, the control drug, cidofovir and ganciclovir have less binding affinity for DNA polymerase of HAdV when compare to that of abacavir and zidovudine. Similarity was observed in the binding of abacavir and zidovudine to HAdV DNA polymerase (ASP742, ALA743, LEU772, ARG773 and VAL776), with that of zidovudine binding to human telomerase reverse transcriptase (TYR137, TYR168, TYR772, MET773, and GLN775). Also, similarity was observed in the binding of cidofovir and ganciclovir to HAdV DNA polymerase.

**TABLE 2:** Molecular docking properties and binding energy score.

SN	Molecular target	UniProt ID	PDB ID	Docking parameters	Binding energy (kcal.mol^-1^)								
					A	B	C	D	E	F	G	H	I
1	Reverse transcriptase/RNaseH (HIV)	Q72547	2jle	Spacing: 0.600	−6.1	−6.0	−6.2	−6.5	−6.1	−7.3			
				Npts: 126 × 126 × 110									
				Center: 7.195 × −40.568 × 34.973									
2	HIV 1 protease (Pol polyprotein)	Q72874	5V4Y	Spacing: 0.372							−6.2		
				Npts: 102 × 100 × 126									
				Center: 8.004 × −21.004 × 6.798									
3	Protein P (HBV-F)	Q05486	Model_Q05486	Spacing: 0.800	−5.7	−5.4							
				Npts: 90 × 70 × 126									
				Center: 129.104 × 119.942 × 126.270									
4	Telomerase reverse transcriptase (Human)	O14746	AF-O14746-F1-model_v4	Spacing: 0.750			−6.2						
				Npts: 126 × 126 × 126									
				Center: 9.587 × 8.454 × −9.024									
5	HLA class I histocompatibility antigen, B-57 alpha chain (Human)	P01889	AF-P01889-F1-model_v4	Spacing: 0.600				−6.3					
				Npts: 126 × 100 × 126									
				Center: 0.273 × 2.144 × −11.704									
6	Purine nucleoside phosphorylase (Human)	P004917	AF-P00491-F1-model_v4	Spacing: 0.500					−7.2				
				Npts: 100 × 96 × 126									
				Center: 1.492 × −0.191 × 0.118									
7	Nuclear receptor subfamily 1 group I member 2 (Human)	O75469	AF-O75469-F1-model_v4	Spacing: 0.750						−8.1	−6.2		
				Npts: 100 × 92 × 126									
				Center: 0.612 × −3.545 × 8.077									
8	DNA polymerase (HAdV hypothetical)	P03261	Model_P03261	Spacing: 0.700			−5.4	−5.8				−5.1	−4.7
				Npts: 126 × 126 × 126									
				Center: 16.866 × 19.442 × 139.025									

A = Lamivudine. B = Tenofovir Disoproxil. C = Zidovudine. D = Abacavir. E = Didanosine. F = Rilpivirine. G = Ritonavir. H = Cidofovir. I = ganciclovir.

**FIGURE 8:**
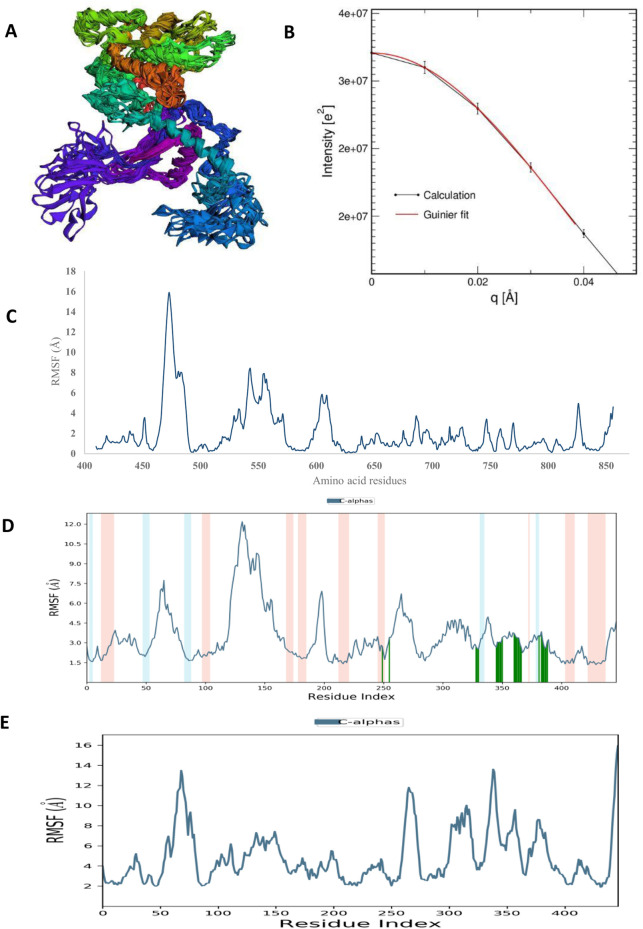
The structural characteristics of HAdV hypothetical protein DNA polymerase (PDB ID: model_P03261): **(A)** CABSFlex2 superimposition of 10 model structures **(B)** SWAXS net intensity Guinier fit. **(C)** Simulated RMSF of free protein using CABSFlex2 server **(D)** Simulated RMSF in the presence of Abacavir using Desmond **(E)** Simulated RMSF in the presence of zidovudine using Desmond.

**FIGURE 9:**
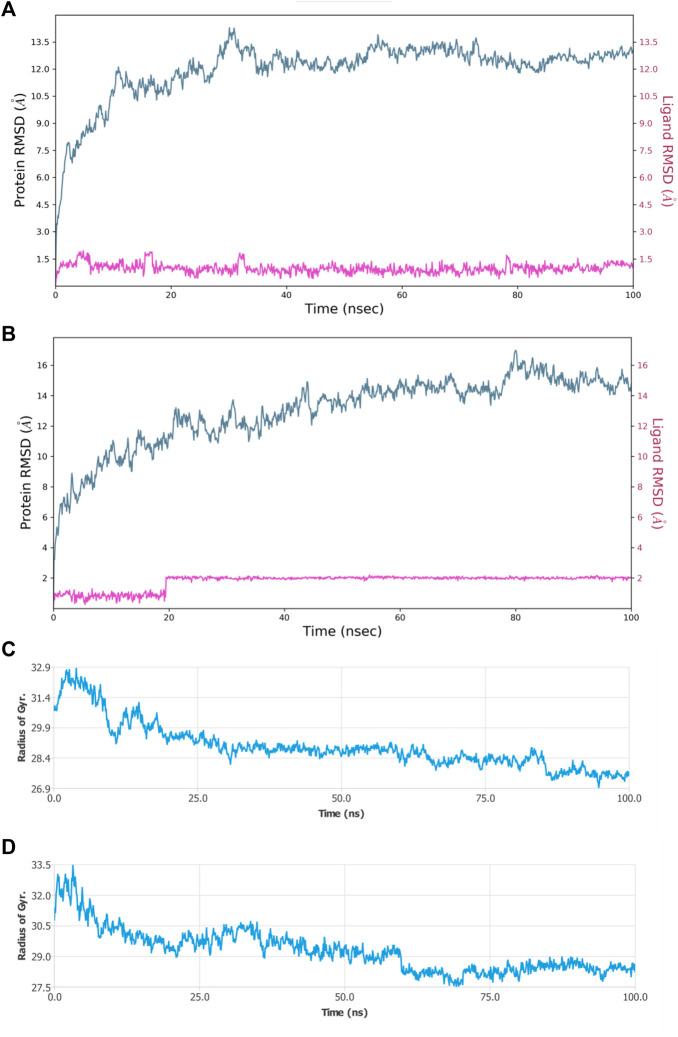
MDS results **(A)** RMSD plot of HAdV DNA pol (PDB ID: model_P03261) in complex with Abacavir **(B)** RMSD plot of HAdV DNA pol (PDB ID: model_P03261) in complex with Zidovudine **(C)** Rg of HAdV DNA pol (PDB ID: model_P03261) in the presence of Abacavir. **(D)** Rg of HAdV DNA pol (PDB ID: model_P03261) in the presence of Zidovudine.

**FIGURE 10:**
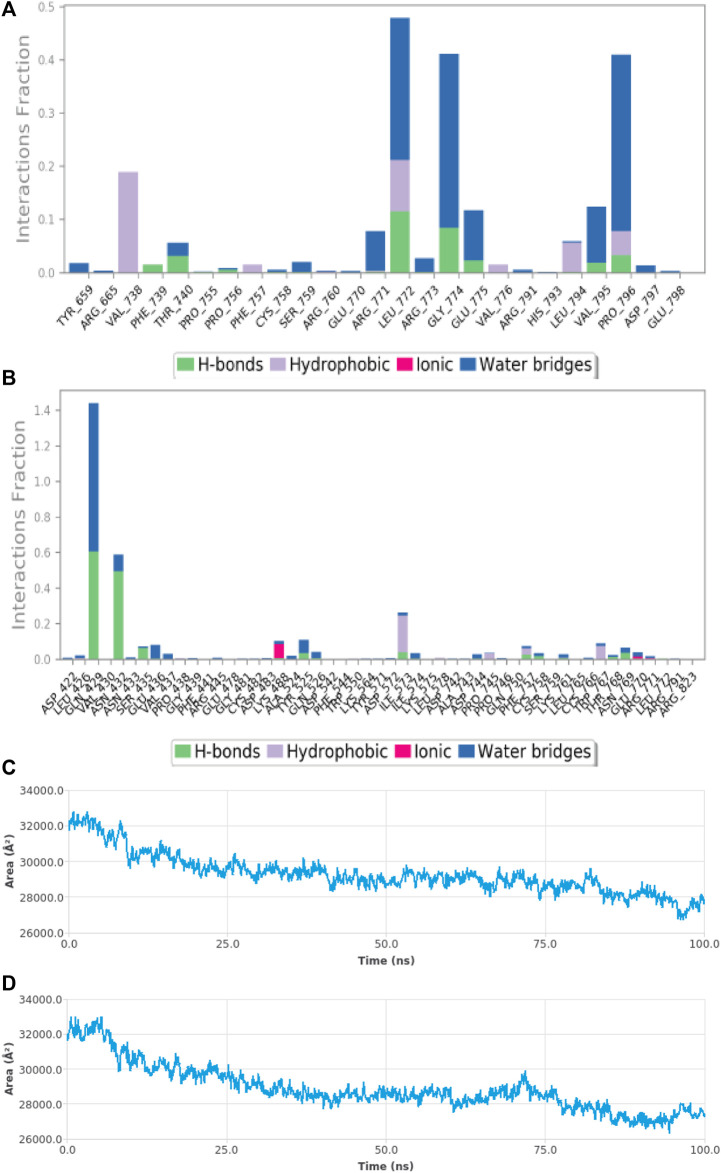
MDS results **(A)** Sub-energy interaction of HAdV DNA pol (PDB ID: model_P03261) in complex with Abacavir. **(B)** Sub-energy interaction of HAdV DNA pol (PDB ID: model_P03261) in complex with Zidovudine. **(C)** SASA of HAdV DNA pol (PDB ID: model_P03261) in the presence of Abacavir. **(D)** SASA of HAdV DNA pol (PDB ID: model_P03261) in the presence of Zidovudine.

**FIGURE 11:**
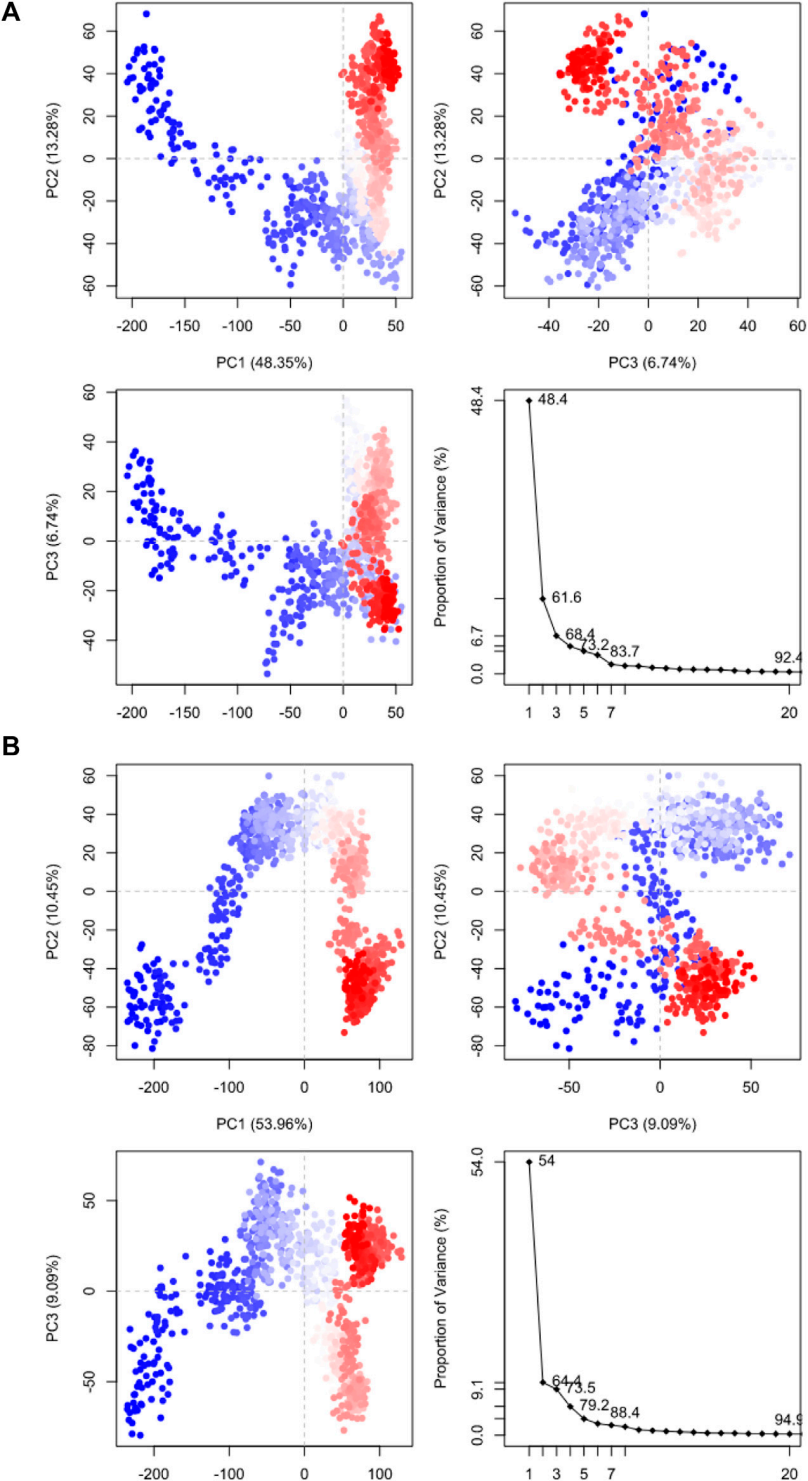
Principal component analysis results of **(A)** HAdV DNA pol (PDB ID: model_P03261) in complex with Abacavir. **(B)** HAdV DNA pol (PDB ID: model_P03261) in complex with Zidovudine.

Molecular dynamic simulations (MDS) were carried out to predict the structural stability of the protein as well as the ligand binding status in the physiological environment. The results of MDS study of the HAdV DNa pol is showed in Figure 8. The results showed radius of gyration (Rg) of 31.98Å (3.198 nm) from SWAXS analysis, also native and the residue wise RMSF value of the free protein and protein bound to the ligands. The residues showing higher peaks correspond to loop regions, as identified from MD trajectories. RMSD values for the protein and ligand in complex are 6 Å and 1 Å respectively, and the Rg of the protein in complex with the ligand is about 6 Å (Figure 9). The results of interactions of various binding forces, SASA and PCA results were indicated in Figure 10 and Figure 11, where the protein SASA values were about 6,000 Å. Val738, Phe739, Leu771, Arg773, Gly774, Leu794 and Val795 were the most interacted amino acid residues of HAdV DNA pol—abacavir complex while Leu429, Asn432, Ile573 and Glu769 were the most interacted amino acid residues of HAdV DNA pol—zidovudine complex. Also, the results of MMGBSA binding energy of protein-ligand interaction showed that HAdV DNA pol—zidovudine complex is higher than that of DNA pol—abacavir complex as shown in Table 3.

**TABLE 3:** Prime MMGBSA binding energy of interaction of HAdV DNA pol with abacavir and zidovudine, before and after molecular dynamics simulation.

Complex	Simulation time (ns)	MMGBSA ΔG^bind^ (kcal.mol^-1^)							
		Total	Coulomb	Covalent	Hbond	Lipo	Packing	Solv_GB	vdW
HAdV DNA pol—abacavir	0	−23.802	−13.922	10.415	−1.230	−8.4176	−0.009	17.363	−28.000
	100	−24.057	−7.729	3.789	−0.043	−8.421	0.000	13.415	−25.067
HAdV DNA pol - zidovudine	0	−32.469	−28.073	0.466	−1.029	−5.817	−2.465	35.649	−31.202
	100	−32.289	−44.422	1.767	−1.002	−4.557	−1.430	48.389	−31.032

Legend: Total: Total energy (Prime energy). Coulomb: Coulomb energy. Covalent: Covalent binding energy. Hbond: Hydrogen bonding energy. Lipo: Lipophilic energy. Packing: Pi-pi packing correction. Solv GB: Generalized Born electrostatic solvation energy. vdW: van der waals energy.

## Discussion

HAdVs have been implicated as infectious agents which are responsible for numerous diseases, including respiratory tract infections, ocular and gastrointestinal tract disorders (Kinchington et al., 2005). The HAdV early regions (E1A, E1B, E2A, E2B, E3 and E4) are the first viral proteins to be expressed during infection (Saha and Parks, 2020). The early E2 region of the HAdV genome consists of two transcription units, E2A encode DNA binding protein (DBP), whereas E2B encode terminal protein precursor (pTP), and DNA polymerase) that are required for viral replication (Robinson et al., 2008). The E2B gene of HAdV encodes DNA polymerase, which plays a key role in the viral genome replication and serves as a potent target for developing antiviral agents (Kumar et al., 2019).

Predicting the structure and function of hypothetical protein provides an opportunity to discover novel potential drug targets (Singh et al., 2022). Computational analysis has shown that two conserved domains; the polymerase/reverse transcriptase (RT) domain, and the C-terminal RNase H (RH) domain, are presence in all P proteins (Xiong and Eickbush, 1990). Previous bioinformatics study has characterized 38 randomly retrieved hypothetical proteins and assessed their physiochemical properties, subcellular localization, and predicted the function of six hypothetical proteins with UniProt IDs: P03269, P03261, P03263, Q83127, Q1L4D7 and I6LEV1 to be DNA terminal protein, DNA polymerase, DNA binding protein, adenovirus E3 region protein CR1 and adenoviral protein L1, respectively (Naveed et al., 2017).

The result of this study showed that antiretroviral drugs mechanism of action is centralized on the simultaneous up-regulating CD4 gene and inhibiting the HAdV DNA polymerase activity by downregulation of hTERT. Telomerase which is highly expressed in the vast majority of human cancers but not in most host tissues, is a unique DNA polymerase that catalyzes the addition of telomeric repeats (T2AG3)_*n*_ to the ends of chromosomes to maintain chromosomal integrity (Mo et al., 2003; Irving et al., 2004). Over 85% of hTERT gene, which codes for the catalytic component of the telomerase ribonucleoprotein complex, are involve in cancers (Meyerson et al., 1997; Irving et al., 2004; Doloff et al., 2008). A research report showed that expression of the E1A gene under the control of the hTERT promoter and subsequently viral replication restricted to tumor cells derived from hepatic and prostate tissues, induced significant tumor reduction and sometimes resulted in complete tumor regression (Irving et al., 2004; Doloff et al., 2008).

A study has shown that adenovirus E3-19k inhibits the phosphorylation of major histocompatibility complex (MHC) class I proteins in the rough endoplasmic reticulum (RER) with ability to bind to HLA molecules, during the infection of a human cell line (Lippe et al., 1991). E3-19K proteins with MHC (class I and class II-restricted) activity binds with high affinity to HLA-A and HLA-B molecules, and counteract activation of CD8^+^ and CD4^+^ T cells (Scheppler et al., 1989; Peek et al., 1994; Li et al., 2016). Thus, vaccines will be unsuitable due to declining of CD4^+^ T cell counts, but antiviral therapies are a promising therapeutic strategy against HAdV infection (Widman et al., 2018).

Several CD4^+^ and CD8^+^ T-cell epitopes from HAdV hexon have been identified and found to be highly conserved among different serotypes (Kumar et al., 2019). The clearance of human adenovirus viremia in patients after allogeneic stem cell transplantation, concurred with advent of a coordinated CD4^+^ and CD8^+^ T-cell response against adenovirus hexon epitopes (Zandvliet et al., 2010). Adenoviruses produce factors that block the synthesis and expression of HLA class I molecules on the cell surface, thereby inhibit the presentation of viral antigens attacked by CD8^+^ T-lymphocytes; and the infected cells acquire increased resistance to interferons and TNF-α (Cook and Radke, 2017). Reduction in the level of surface-expressed HLA class I cause Immunosuppression and it has been reported to occur during infection by several viruses including HAdV through mechanism that differs from inhibition of host cell protein synthesis. This is possible by downregulation of the surface expression of the T cell specific CD3 complex and HLA class I, which resulted in impairment of the presentation of viral peptides to the immune system and thus prevent the initiation of cell-mediated immunity to the virus (Boshkov et al., 1992; Peek et al., 1994).

Insight on the DEGs during adenovirus infection could provide rich clues to understanding the host’s response to adenovirus infection at the different stages of their replicative cycle. This study showed the metabolism genes involved in ARVD, which include kinases (CDK1, CDK2, CDK4, HIPK, DNAPK, MAPK3 and MAPK14) and transcription factors (MYC, HNF4A, MAX, TP63, GATA1, REST, PPARD, FOXA2, and NFE2L2), which must be well regulated in order to inhibit HAdV patho-mechanism. A study on HBV replication in human hepatic cells has published that FOXA1 and FOXA2 suppressed the expression of nuclear hormone receptors including PPARα, HNF4α, and RXRα (Okumura et al., 2015). A study has identified CD24, CD44, TP63, AHR, MAP3K14, RARB, PIMI, NEDD9, and few others, to be DEGs suppressed in HAdV-6 and HAdV-12, while CBX7 is an activated DEGs found in HAdV-12 (Wang et al., 2018). AHR is one of the DEG found involved in cell cycle control in HAdVs while CDK2 is one of the key regulators for the progression from G1 to the S phase, and c-Myc:Max is one of the consensus transcription factor expressed in HAdVs (Zhao et al., 2019). In fowl adenovirus serotype 4 (FAdV-4 strain GX-1), FABP3 and PPARG were reported to be upregulated DEGs at 21dpi of KEGG pathway (Ren et al., 2019).

In respect to HAdV antivirals molecular targets, the CDKs have been found promising because they regulate key aspects of the replicative cycles of a number of DNA and RNA viruses. Specifically, CDK2 was shown to promote the replication of HAdV, while CDK9 has been suggested to enhance transcription of the HAdV E1A gene (Dodge et al., 2021). MYC is a transcription factor that regulates energy metabolism through direct activation of metabolic genes, thus the ARVDS mechanism would be to deactivates MYC in the virus-infected patients. During hepatitis C virus (HCV) infection, upregulation of MYC was shown to suppresses the expression of N-Myc downstream-regulated gene 1 (*NDRG1*), and lead to decreased expression of NDRG1-specific kinase serum/glucocorticoid-regulated kinase 1 (SGK1) (Schweitzer et al., 2018).

Moreover, several DEGs were found downregulated during the progression of an adenovirus infection including DEGs involved in cell cycle and proliferation (MYC, SGK), in immune and stress response (NFE2L2, MAP2K3), in cellular structure and cell communication (CD44, KIF23), but about 50% of DEGs involved in metabolism (e.g., FKBP4, HSD17B2) were upregulated (Granberg et al., 2005). Insightfully, HAdV could activates MYC gene of the host to upregulates the expression of host cell glucose metabolic enzymes (specifically, hexokinase 2 and phosphofructokinase 1) through a process that mimics Warburg effect in cancer, to promote biosynthesis of nucleotide from glucose intermediates and ensure sustainable environments for its replication in primary lung epithelial cells (Thai et al., 2014). Thus, downregulation of MYC by ARVDS combination therapy will trigger expression of NDRG1, which inhibits HCV and HAdV replication at the stage of viral assembly. The results of this study showed that rilpivirine and ritonavir targets the human nuclear receptor subfamily 1 group I member 2 (NR1I2). Also, nuclear receptor subfamily 4, group A, member 1 (NR4A1) have been reported to be among the DEGs that showed decrease expression during the progression of an adenovirus infection (Granberg et al., 2005).

The result of this study showed that cidofovir and ganciclovir have less binding affinity for DNA polymerase of HAdV when compare to zidovudine and abacavir. Curated information on DrugBank database showed that cidofovir (DB00369) is an inhibitor of DNA polymerase catalytic subunit (UniProt ID: P08547) of human herpesvirus 5 (HHV-5) in cytomegalovirus (CMV) family; Ganciclovir (DB01004) is an inhibitor of DNA polymerase catalytic subunit (UniProt ID: P04293) of HHV-1. Ganciclovir is of little value as a HAdV therapeutic due to the fact that it requires activation by a viral thymidine kinase to be most effective, which is lacking in HAdV (Dodge et al., 2021). However, study has shown that HAdV co-infection with human CMV improved anti-HAdV activity of ganciclovir, due to CMV viral kinase-mediated activation of ganciclovir (Aguilar-Guisado et al., 2020).

Abacavir and Zidovudine inhibit the HIV-1 reverse transcriptase enzyme competitively and act as a chain terminator of DNA synthesis. Abacavir is a carbocyclic synthetic nucleoside analogue and an antiviral agent. Intracellularly, abacavir is phosphorylated by cellular enzymes to the active metabolite carbovir triphosphate, an analogue of deoxyguanosine-5′-triphosphate (dGTP). Carbovir triphosphate inhibits the activity of HIV-1 reverse transcriptase (RT) both by competing with the natural substrate dGTP and by its incorporation into viral DNA. Viral DNA growth is terminated because the incorporated nucleotide lacks a 3′-OH group, which is needed to form the 5′to 3′phosphodiester linkage essential for DNA chain elongation (De Clercq, 2002). Zidovudine, a structural analog of thymidine, is a prodrug that is phosphorylated to produce active metabolite, zidovudine triphosphate (ZDV-TP), which inhibits the activity of HIV-1 reverse transcriptase (RT) via DNA chain termination by competing with the natural substrate dGTP and incorporates itself into viral DNA. zidovudine is also a weak inhibitor of cellular DNA polymerase α and γ (De Clercq, 2010; Leeansyah et al., 2013). A study has shown that zidovudine could inhibit human telomerase reverse transcriptase (hTERT) (Leeansyah et al., 2013), while abacavir could target human HLA class I histocompatibility antigen, B-57 alpha chain protein (Yang et al., 2009).

Computational approaches such as *in silico* molecular docking and molecular dynamic simulation, provide opportunities for target discovery and drug repositioning are useful in modern pharmaceutical processes. Drug repurposing or repositioning is now a significantly relevant strategy to circumvent the roadblocks in novel drug discovery (Pushpakom et al., 2019; Dodge et al., 2021).

Molecular dynamics simulation (MDS) was performed to understand the structural and conformational changes in the protein and protein-ligand complex. Maestro-Desmond was used for the MSD in this study, and it is an interoperable tool built for biomolecular (protein, RNA and DNA) simulation (Schrodinger, 2018). RMSD plots is used to indicate whether there are no huge changes in the Cα backbone of the protein. The smaller amount of difference in the value of Rg as well as RMSD indicate firmly folded protein (Saini et al., 2021). Radius of gyration (Rg) indicates the overall compactness of the protein during the molecular dynamics. It is the distance between the center of mass of all atoms of protein and its terminal in a particular time interval. Low RMSF values of binding site residues indicate the stability of ligand binding to the protein. The MMGBSA was used to determine the binding energy from the free energies of the reactants and product of the reaction (Kollman et al., 2000).

## Conclusion

The results of this study pinpointed abacavir and zidovudine as potential repurposing FDA drugs for inhibiting HAdV DNA polymerase. Combination therapy for treating multisystemic diseases such as neurodegeneration (i.e., amyotrophic lateral sclerosis) and parasitic (i.e., HIV-AIDS, malaria) illnesses could be well implemented for HAdV treatment. Combined use of existing approved fosters synergistic efficacy, ameliorate resistance to single drug, and improved tolerance based on complementarity in the pharmacokinetics and pharmacodynamics of the drug. Future work will be necessary to validate the combine therapy of abacavir, zidovudine and other existing investigational drug for treating HAdV infection such as brincidofovir, tazarotene, and verdinexor.

## Data Availability

The original contributions presented in the study are included in the article/Supplementary Material, further inquiries can be directed to the corresponding author.
