# Supplementary material for: Human adenovirus DNA polymerase is evolutionarily and functionally associated with human telomerase reverse transcriptase based on in silico molecular characterization that implicate abacavir and zidovudine
Source: Front Bioinform. 2023 Jun 7;3:1123307. doi: 10.3389/fbinf.2023.1123307 (PMC10282644; doi:10.3389/fbinf.2023.1123307)
Supplement: Supplementary file 1 [file DataSheet1.DOCX]

| **Table S1: List of 38 Hypothetical Proteins of Human Adenovirus along with their Uniprot ID, corresponding genome and protein length** | | | | | | | | |
| --- | --- | --- | --- | --- | --- | --- | --- | --- |
| Sr No | **UniProt ID** | **HAdV** | **Protein Length** | **Molecular Weight** | **Isoelectric point (pI)** | **Net Charge** | **A280 Extinction coefficients 1mg/ml (M^-1^.cm^-1^)** | **Improbability of expression in inclusion bodies** |
| 01 | P03269 | HAdV-2 | 671 | 76542.76 | 6.2214 | -2.5 | 0.984 | 0.566 |
| **02** | **P03261** | **HAdV-2** | **1198** | **135669.98** | **7.4118** | **14.5** | **1.313** | **0.687** |
| 03 | P03263 | HAdV-2 | 145 | 16102.48 | 6.3753 | -0.5 | 1.644 | 0.701 |
| 04 | Q83127 | HAdV-7 | 179 | 20020.61 | 9.4806 | 10.5 | 1.019 | 0.787 |
| 05 | P03287 | HAdV-2 | 106 | 11668.29 | 12.6799 | 22.0 | 0.599 | 0.854 |
| 06 | P03289 | HAdV-2 | 112 | 12811.02 | 12.9339 | 25.5 | 0.662 | 0.979 |
| 07 | P03294 | HAdV-2 | 121 | 12755.76 | 11.9217 | 13.5 | 0.979 | 0.643 |
| 08 | P03292 | HAdV-2 | 168 | 17703.00 | 12.7532 | 24.5 | 0.932 | 0.800 |
| 09 | P03291 | HAdV-2 | 215 | 22787.75 | 12.5345 | 32.5 | 1.096 | 0.822 |
| 10 | P03293 | HAdV-2 | 137 | 14356.12 | 10.5721 | 9.0 | 1.636 | 0.954 |
| 11 | Q1L4D7 | Human mastadenovirus B | 146 | 16346.57 | 4.7464 | -5.5 | 1.465 | 0.718 |
| 12 | I6LEV1 | Human mastadenovirus B | 146 | 16404.61 | 4.6215 | -6.5 | 1.460 | 0.777 |
| 13 | E1ARQ3 | Human mastadenovirus C | 121 | 12739.72 | 11.9217 | 13.5 | 0.980 | 0.672 |
| 14 | Q4JEP5 | HAdV-7 | 81 | 8779.87 | 12.2193 | 15.5 | 0.339 | 0.729 |
| 15 | Q5EY75 | HAdV-7 | 114 | 12607.02 | 12.1107 | 19.5 | 0.355 | 0.590 |
| 16 | Q2KS67 | HAdV-7 | 173 | 19013.90 | 12.0259 | 19.5 | 2.392 | 0.853 |
| 17 | E1U5M6 | HAdV-6 | 112 | 12777.00 | 12.9339 | 25.5 | 0.664 | 0.979 |
| 18 | E1U5N2 | HAdV-6 | 176 | 18702.56 | 12.1123 | 23.0 | 0.747 | 0.636 |
| 19 | A0A0B4SHT8 | Human adenovirus 21 | 104 | 11513.51 | 10.6914 | 13.5 | 2.040 | 0.551 |
| 20 | A0A0B4SJJ5 | Human adenovirus 21 | 114 | 12578.97 | 12.1107 | 19.5 | 0.355 | 0.590 |
| 21 | A0A0B4SI61 | Human adenovirus 21 | 173 | 18939.90 | 12.1645 | 21.5 | 2.111 | 0.801 |
| 22 | A0A0B4SHQ0 | Human adenovirus 21 | 114 | 12605.05 | 12.1107 | 19.5 | 0.355 | 0.623 |
| 23 | Q3ZKV3 | Human adenovirus 50 | 104 | 11497.51 | 10.6914 | 13.5 | 2.043 | 0.589 |
| 24 | Q5EY73 | HAdV-7 | 133 | 14556.41 | 4.7319 | -8.0 | 0.102 | 0.890 |
| 25 | Q2KS66 | HAdV-7 | 95 | 10357.56 | 3.9479 | -13.5 | 0.144 | 0.979 |
| 26 | I1V173 | HAdV-7 | 91 | 9693.08 | 6.6883 | 1.0 | 0.307 | 0.660 |
| 27 | Q2KS62 | HAdV-7 | 133 | 14557.55 | 4.8299 | -6.5 | 0.205 | 0.830 |
| 28 | A0A0B4SIA5 | Human adenovirus 21 | 91 | 9744.05 | 6.2004 | -1.5 | 0.153 | 0.574 |
| 29 | A0A0B4SGV2 | Human adenovirus 21 | 133 | 14546.43 | 4.7319 | -8.0 | 0.102 | 0.916 |
| 30 | A0A0B4SIU9 | Human adenovirus 21 | 133 | 14545.44 | 4.8498 | -7.0 | 0.102 | 0.877 |
| 31 | Q2KS78 | Human adenovirus 21 | 95 | 10394.62 | 3.8629 | -14.0 | 0.143 | 0.979 |
| 32 | Q2KSC0 | Human adenovirus 21 | 91 | 9745.04 | 5.9793 | -2.5 | 0.153 | 0.689 |
| 33 | A0A0B4SH32 | Human adenovirus 21 | 106 | 11433.21 | 12.4222 | 12.5 | 1.092 | 0.861 |
| 34 | Q3ZKV7 | Human adenovirus 50 | 173 | 18884.67 | 12.0259 | 19.5 | 2.118 | 0.865 |
| 35 | Q3ZKV4 | Human adenovirus 50 | 133 | 14624.55 | 5.0104 | -6.5 | 0.102 | 0.877 |
| 36 | Q3ZKV2 | Human adenovirus 50 | 91 | 9745.04 | 5.9793 | -2.5 | 0.153 | 0.689 |
| 37 | A6MLW9 | Human mastadenovirus E | 106 | 11494.34 | 13.5100 | 17.0 | 0.608 | 0.506 |
| 38 | E1U5M8 | HAdV-6 | 215 | 22815.76 | 12.5594 | 32.5 | 1.095 | 0.822 |
| **X** | **O14746** | **Human sapien TERT** | **1132** | **126996.72** | **11.0778** | **103.0** | **1.108** | **0.609** |
